# The Practical Value of Xpert MTB/RIF Ultra for Diagnosis of Pulmonary Tuberculosis in a High Tuberculosis Burden Setting: a Prospective Multicenter Diagnostic Accuracy Study

**DOI:** 10.1128/spectrum.00949-22

**Published:** 2022-07-25

**Authors:** Guirong Wang, Mingxiang Huang, Hui Jing, Junnan Jia, Lingling Dong, Liping Zhao, Fen Wang, Yi Xue, Yunfeng Deng, Guanglu Jiang, Hairong Huang

**Affiliations:** a National Clinical Laboratory on Tuberculosis, Beijing Key Laboratory for Drug-Resistant Tuberculosis Research, Beijing Chest Hospital, Capital Medical University, Beijing Tuberculosis and Thoracic Tumor Institute, Beijing, People’s Republic of China; b Fuzhou Pulmonary Hospital of Fujian, Fuzhou, People’s Republic of China; c Katharine Hsu International Research Center of Human Infectious Diseases, Shandong Public Health Clinical Center, Jinan, Shandong, People’s Republic of China; Johns Hopkins University School of Medicine

**Keywords:** tuberculosis, pulmonary, Xpert Ultra, trace, specificity

## Abstract

Due to the probability of decreased specificity, the practical value of performing the Xpert MTB/RIF Ultra (Xpert Ultra) assay over the Xpert assay for diagnosing pulmonary tuberculosis (TB) and rifampicin (RIF) resistance in a high TB burden setting was evaluated. Participants were recruited consecutively in three tertiary hospitals in China and allocated to the TB case detection and/or rifampicin (RIF) resistance detection group. Each sputum specimen was subjected to smear, MGIT960 liquid culture, and Xpert, and Xpert Ultra assay in parallel. Drug susceptibility testing was conducted for all recovered isolates in the RIF resistance detection group. In total, 1,079 patients were recruited to the case detection group and 450 to the RIF resistance detection group. Xpert Ultra had higher sensitivity than Xpert (92.26%, 322/349 versus 89.40%, 312/349; *P* = 0.006), whereas the most prominent increase was identified in the smear-negative patients (83.70% versus 78.52%; *P* = 0.039). The specificity of Xpert Ultra was slightly lower than that of Xpert (96.30%, 495/514 versus 98.25%, 505/514; *P* = 0.055). Reclassifying trace results as negative resulted in a 4.01% loss of sensitivity (from 92.26% to 88.25%) accompanied by a 1.37% gain in specificity (from 96.30% to 97.67%). Both the sensitivity (97.64% versus 99.21%, *P* = 0.313) and specificity (96.90% versus 97.21%, *P* = 0.816) of Xpert Ultra and Xpert for detection RIF resistance were comparable. In conclusion, Xpert Ultra could improve the diagnosis of smear-negative pulmonary TB in contrast to the Xpert assay. A high percentage of TB history did not significantly decrease the specificity of the test, which supports the potential role of Xpert Ultra as an initial diagnostic tool for TB.

**IMPORTANCE** Xpert Ultra is more sensitive than Xpert, especially in smear-negative TB. A high percentage of TB history in the non-TB population did not significantly affect the reliability of the assay, which supports the potential role of Xpert Ultra as an initial diagnostic tool for TB.

## INTRODUCTION

Tuberculosis (TB) is a leading cause of infectious disease-related deaths. Globally, an estimated 9.9 million people fell ill with TB in 2020 (1). Of the 4.8 million people diagnosed with pulmonary TB worldwide in 2020, 59% were bacteriologically confirmed (1). In addition, 71% (2.1/3.0 million) of bacteriologically confirmed pulmonary TB patients were tested for rifampicin (RIF) resistance (1). The considerable detection gap was mainly caused by shortage and incapability of diagnostics, especially in high TB burden countries. Therefore, highly sensitive, rapid, and accessible diagnostics are persistently needed.

The WHO recommended the Xpert MTB/RIF assay (Xpert) (Cepheid Inc., Sunnyvale, CA, USA) as the initial test for pulmonary TB in 2010 (2). Xpert shows excellent sensitivity (~98%) in diagnosing pulmonary TB with smear-positive sputum; however, the sensitivities of Xpert in pulmonary TB with smear-negative sputum (67%), in HIV-positive participants (81%), and in children (62%) are considered suboptimal (3). Furthermore, it has also been reported that Xpert occasionally gives false-positive results when used for detecting RIF resistance due to the silent mutations in the *rpoB* gene or samples with very low bacterial loads (4). Consequently, the next-generation cartridge, Xpert MTB/RIF Ultra (Xpert Ultra) (Cepheid Inc., Sunnyvale, CA, USA), was developed and expected to improve the diagnosis of TB and RIF resistance and was recommended by the WHO in March 2017 (5). Consistent outcomes from different studies demonstrated higher sensitivity but lower specificity of Xpert Ultra compared to Xpert, and false-positive results were often obtained from patients who had TB histories (6–9). Because the compromised specificity of the Xpert Ultra assay is largely based on trace results, its interpretation and how to translate it into daily clinical practice remain controversial (10, 11). As country-level tuberculosis incidence rates seem to affect the specificity of Xpert Ultra, further research in high burden settings is needed to clarify the implications of the trade-off between increased sensitivity and decreased specificity.

Previous on-site evaluation studies of Xpert Ultra from China have mainly focused on extrapulmonary tuberculosis specimens or pulmonary TB from a single center with very small sample size (12, 13). Studies including multiple centers and a large-scale pulmonary TB sample size have never been performed in China, which is a high tuberculosis and multidrug-resistant tuberculosis burden setting. Evaluation of the performance and potential application of this advanced diagnostic in the real world is of high importance.

## RESULTS

### Patient characteristics.

**FIG 1:**
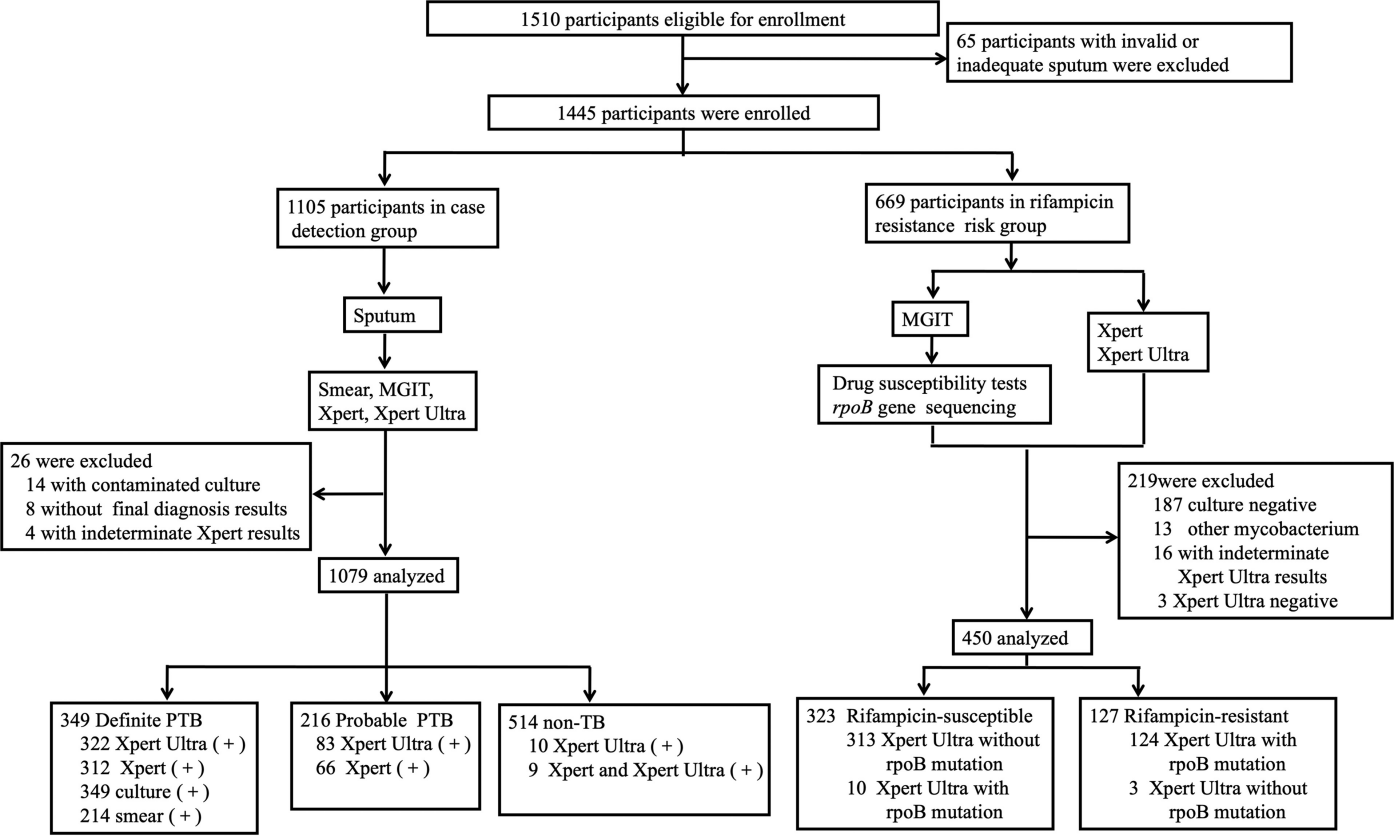
Recruitment and diagnostic classification of the participants

In total, 1,445 participants were enrolled at the three sites (Fig. 1). Among the 1,105 participants in the case detection group, 26 cases were excluded and 1,079 patients were included in the analyses, which included 349 definite pulmonary TB (32.34%), 216 probable pulmonary TB (20.02%), and 514 non-TB (47.64%) cases. In contrast, among the 669 participants in the RIF resistance detection group, 219 cases were excluded and the final sample size for analysis was 450 patients, which included 323 RIF-susceptible pulmonary TB (71.80%) and 127 RIF-resistant pulmonary TB (28.22%) cases. All patients were HIV-uninfected. The median age was 57 years, with women making up about one-third (29.44%) of the participants. Basic characteristics stratified by hospital are shown in Table 1.

**TABLE 1:** Characteristics of study participants stratified by hospital

Characteristics	Overall	Beijing chest hospital	Shandong provincial chest hospital	Fuzhou pulmonary hospital of Fujian
Demographic or clinical characteristics				
Age, median (range), yr	57 (7–95)	56 (15–95)	51 (7–91)	57 (15–93)
Gender (Male/Female)	973/406	273/105	328/184	372/117
HIV infection	0/1379	0/378	0/512	0/489
History of tuberculosis	68/1379 (4.93)	13/378 (3.44)	11/512 (2.15)	44/489 (9.00)
Enrolment group				
Case detection group	1079	217	443	419
Rifampicin resistance risk group	669	274	217	178

### Performance of Xpert Ultra in pulmonary TB diagnosis.

Against the mycobacterial culture reference standard, the direct head-to-head comparative accuracy for Mtb detection showed that Xpert Ultra had higher sensitivity than Xpert (92.26%, 322/349 versus 89.40%, 312/349; *P* = 0.006). According to the analysis after stratification by the smear outcomes, Xpert Ultra showed significantly higher sensitivity than Xpert among culture-positive smear-negative sputum (83.70%, 113/135 versus 78.52%, 106/135; *P* = 0.039). Both Xpert Ultra (97.66%, 209/214) and Xpert (96.26%, 206/214) showed excellent performance in diagnosing pulmonary TB from culture-positive smear-positive sputum (Table 2). When Xpert Ultra outcomes were integrated for diagnosis, 83 of the 216 (38.43%) probable pulmonary TB cases were found to have bacteriologic evidence.

**TABLE 2:** Performance of Xpert and Xpert Ultra for diagnosing pulmonary tuberculosis

	Xpert	Xpert Ultra	*P* value
Sensitivity			
Definite pulmonary TB	312/349 (89.40)	322/349 (92.26)	0.006
Culture positive smear positive	206/214 (96.26)	209/214 (97.66)	0.250
Culture positive smear negative	106/135 (78.52)	113/135 (83.70)	0.039
Probable pulmonary TB	66/216 (30.56)	83/216 (38.43)	<0.001
Specificity	505/514 (98.25)	495/514 (96.30)	0.055
Positive predictive value	312/321 (97.20)	322/341 (94.43)	0.077
Negative predictive value	505/542 (93.17)	495/522 (94.83)	0.257

The specificity of Xpert Ultra was slightly lower than Xpert (96.30%, 495/514 versus 98.25%, 505/514; *P* = 0.055), although the difference was not statistically significant (Table 2). Among the 19 patients with false-positive Xpert Ultra outcomes, 5 had a known pulmonary TB history, whereas another 2 had NTM infections (one Mycobacterium intracellular infection and one Mycobacterium *kansassi* infection), 1 had lung cancer, and 11 had pneumonia.

### Performance of trace semiquantitation reclassification.

Twenty-eight pulmonary TB cases and 7 non-TB patients produced trace results with Xpert Ultra. To further elucidate the significance of trace, these cases were assigned to different categories. (i) True-positive (57.14%, 20/35): samples with positive outcome by any of smear (2 cases), culture (14 cases) or Xpert (15 cases), or by other tests during follow up (1 case). (ii) Possible true-positive (22.86%, 8/35): samples collected from probable pulmonary TB patients without other bacteriological evidence, and (iii) False-positive (20.00%, 7/35): 7 patients with excluded or improbable diagnosis of TB, including 3 with history of TB.

An additional analysis was conducted to assess the effect of reclassifying trace results as negative on Xpert Ultra test performance. This resulted in a 4.01% loss of sensitivity (from 92.26% to 88.25%) accompanied by a 1.37% gain in specificity (from 96.30% to 97.67%). However, there was a greater loss in sensitivity in the smear-negative pulmonary TB, where sensitivity dropped by 8.89% (from 83.70% to 74.81%).

### Performance of Xpert Ultra in detecting RIF resistance.

Four hundred fifty cases produced phenotypic DST outcomes and eligible Xpert and Xpert Ultra RIF resistance results. Against the reference standards of phenotypic DST, both the sensitivity (97.64%, 124/127 versus 99.21%, 126/127; *P* = 0.313) and specificity (96.90%, 313/323 versus 97.21%, 314/323; *P* = 0.816) of Xpert Ultra and Xpert for detecting RIF resistance were comparable.

Thirteen participants produced discordant RIF drug susceptibility testing results among Xpert, Xpert Ultra, and phenotypic DST. The *rpoB* gene was sequenced to elucidate the RIF susceptibility status. Notably, resistance reported by Xpert or Xpert Ultra was always accompanied with a mutation in the target sequence (Table 3). CTG533CCG (38.46%, 5/13) was the most frequently observed mutation; the second most frequently observed mutation was CTG511CCG (21.43%, 3/14). Furthermore, one specimen reported as phenotypic susceptible but RIF resistant by both Xpert and Xpert Ultra actually had a synonymous mutation at codon 517 (CAG→CAA). According to the sequencing data of the specimens with RIF resistance by Xpert Ultra but RIF susceptible by phenotypic DST, specificity of 98.74% (313/317) for Xpert Ultra was obtained.

**TABLE 3:** Details for participants with discordant rifampin drug susceptibility results by Xpert, Xpert Ultra, and phenotypic drug susceptibility testing^*a*^

Participant ID	Site	Rifampin phenotypic DST result	Xpert rifampin result	Xpert Ultra rifampin result	rpoB gene mutation
151504	Beijing	S	R	R	CAG517CAA
147767	Beijing	S	R	R	CTG533CCG
151202	Beijing	S	R	R	CTG511CCG
252826	Shandong	S	R	R	CTG511CCG
512850	Shandong	S	R	R	CTG533CCG
513668	Shandong	S	R	R	CTG533CCG
261668	Shandong	S	R	R	CTG533CCG
192104	Shandong	S	S	R	CAC526AAC
512834	Shandong	S	R	R	CTG533CCG
512949	Shandong	S	R	R	CTG511CCG
133245	Beijing	R	R	S	GAC516GGC
2001467	Fujian	R	R	S	TCG531TTG
1406777	Fujian	R	R	S	Wild type

*^a^*S, susceptible; R, resistant.

## DISCUSSION

Xpert Ultra has been reengineered to improve diagnostic performance with a lower analytic limit of detection. Although slightly increased sensitivity in detection was acquired with sputum, a decrease in specificity raises concerns about its practical value (6, 7). As the compromised specificity of Xpert Ultra is closely related with the TB history of the subject, different TB prevalence rates would affect Xpert Ultra’s performance in different countries. Hence, the performance of Xpert Ultra was compared head to head with its first-generation counterpart, i.e., Xpert assay, in this multicentered study in China.

Among the 349 definite pulmonary TB patients, Xpert Ultra was only 2.86% (92.26% versus 89.40%) more sensitive than Xpert overall and 5.18% (78.52% versus 83.70%) more sensitive among smear-negative pulmonary TB cases. Our results were in line with previous reports, which showed that using culture as the gold standard the pooled sensitivity of Xpert Ultra in diagnosing pulmonary TB was 84%–91% versus 69%–85% pooled sensitivity for Xpert (9, 14, 15). These results indicate that Xpert Ultra is highly beneficial for diagnosing paucibacillary pulmonary TB patients. Several studies also reported that Xpert Ultra significantly improved the diagnosis of extrapulmonary TB and childhood TB with paucibacillary features (12, 16–18), while comparable specificity was acquired in contrast to the Xpert assay.

Xpert Ultra has an additional category called “trace” in contrast to Xpert. The “trace” category is mainly responsible for the improvement in the limit of detection and reduced specificity of Xpert Ultra. However, it is critical to appropriately interpret the trace readout in clinical practice in order to take advantage of the increased sensitivity and avoid false-positive outcomes. Reclassification of trace results as negative in this study resulted in a 1.37% gain in specificity (from 96.30% to 97.67%); however, the sensitivity in smear-negative pulmonary TB also dropped by 8.89% (from 83.70% to 74.81%). Berhanu et al. (19) reported that reclassification of trace results caused 5.6% loss of sensitivity in the smear-negative group. Dorman et al. (6) showed that the reduction in sensitivity from excluding the trace readout was almost 9% in smear-negative culture-positive persons. Thus, if trace results were reclassified to elevate specificity, Xpert Ultra would lose its sensitivity-associated benefit over Xpert. We have summarized the trace-positive rate of several previous reports (Table 4), which show that trace-positive results are relatively infrequent (<6%) in non-TB patients, except for those with a history of TB treatment within 2 years (15.32%). Furthermore, more than half of the trace outcomes were confirmed by other tests (57.14%, 20/35) in this study. In a highly notable event, one “non-TB” patient, who yielded trace outcome for Xpert Ultra and very low positive outcome for Xpert assay, produced positive molecular outcome during follow-up and was subsequently reclassified as a TB patient. Overall, the influence of TB burden on specificity of Xpert Ultra in China was lower than what we had predicted. Our findings support the potential role of Xpert Ultra as the initial diagnostic tool for pulmonary TB.

**TABLE 4:** Xpert Ultra trace-positive rate of several studies

Author	Year	Country	Sample size	Sample type	Culture-positive (%)	Trace-positive rate (%)		
						Total	TB	Non-TB
Dorman SE, et al. (6)	2018	South Africa, Uganda, Kenya, India, China, Georgia, Belarus, Brazil	1,439	Sputum	32.11 (462/1439)	2.22 (32/1439)	2.81 (13/462)	1.94 (19/977)
Berhanu RH, et al. (19)	2018	Johannesburg, South Africa	237	Sputum	23.63 (56/237)	2.53 (6/237)	1.79 (1/56)	2.76 (5/181)
Opota O, et al. (21)	2019	Switzerland	196	Respiratory sample	23.98 (47/196)	5/196	8.51 (4/47)	0 (0/149)
Mishra H, et al. (7)	2020	South Africa	239	Sputum	30.13 (72/239)	5.44 (13/239)	5.56 (4/72)	5.39 (9/167)
Mishra H, et al. (7)	2020	South Africa	168	Sputum^*a*^	26.19 (44/168)	12.50 (21/168)	4.55 (2/44)	15.32 (19/124)
Esmail A, et al. (11)	2020	South Africa	268	Sputum	62.69 (168/268)	3.36 (9/268)	3.57 (6/168)	3.00 (3/100)
Andama A, et al. (24)	2021	Uganda	698	Sputum		3.01 (21/698）	4.76 (16/336)	1.38 (5/362)
Zhang P, et al. (13)	2021	China	99	Bronchoalveolar lavage	25.25 (25/99)	5.95 (5/99)		
Our study	2021	China	1,079	Sputum	32.34 (349/1079)	3.23 (35/1079)	4.79 (27/564)	1.55 (8/155)

*^a^*Patients with presumptive tuberculosis and recent previous tuberculosis (≤2 years).

Although the decreased specificity of Xpert Ultra is a noteworthy concern for its application in diagnostics in high TB burden settings, we did not observe significantly lower specificity for it compared with Xpert assay (96.30% versus 98.25%, *P* = 0.055). In addition, despite the fact that 15.95% (82/514) of the non-TB participants had a known TB history, only three of them yielded trace outcomes. A majority of the patients had a history of TB extending beyond 2 years; hence, it is plausible that this is the main reason that TB history did not significantly affect the specificity of Xpert Ultra in this study. Therefore, our study supports the practical value of performing Xpert Ultra in China.

Multidrug-resistant TB continues to remain as a concern globally. Xpert Ultra was developed to improve the specificity in detection of RIF resistance; however, we did not observe this improvement over Xpert assay in this study. Here, Xpert Ultra and Xpert displayed comparable sensitivity (97.64% versus 99.21%) and specificity (96.90% V.S. 97.21%) for the detection of RIF resistance, consistent with other reports (20, 21). This could be explained by the fact that the increased sensitivity of Xpert Ultra mainly gives credit to the trace semiqualification outcome target IS6110/IS1083, which has no relation with RIF resistance.

Based on ours and other studies, we suggest two different strategies for the application of Xpert Ultra, considering the cost of Xpert Ultra on the market. When the price of Xpert Ultra assay is similar to that of Xpert assay, Xpert Ultra could be used as a surrogate to the Xpert assay as the initial TB diagnostic test. On the other hand, if the cost of Xpert Ultra assay is obviously higher than Xpert assay, we suggest that Xpert Ultra be used as the initial diagnostic test for paucibacillary TB, such as extrapulmonary TB and childhood TB, while for a pulmonary TB suspect with a negative initial Xpert assay, an additional Xpert Ultra test could be performed to improve case finding.

A strength of our study is that it is a prospective multicenter study with a large number of consecutively enrolled participants in a clinical routine setting. The study population is thus more likely to be representative of the true test population in a high TB prevalence country. But the study’s limitations should also be noted. First, the realistic TB history prevalence rate in the non-TB group is not known. Some participants might have self-recovered undiagnosed TB or have a previously unknown history of TB, which happens frequently in high TB burden settings. However, these possibilities, together with that of the people with known TB history, were evaluated as a whole in this study. Second, only smear-positive sputa were recruited in the RIF resistance detection group. Smear-negative samples are a major source of false RIF resistant results of the Xpert assay. No improvement in specificity of RIF resistance detection of Xpert Ultra was observed in this study, which might relate to the exclusion of smear-negative samples.

In conclusion, Xpert Ultra is more sensitive than Xpert, especially in smear-negative pulmonary TB. A high percentage of TB history in the non-TB population did not significantly affect the reliability of the test, which supports the potential role of Xpert Ultra as an initial diagnostic tool for TB. If the cost of Xpert Ultra assay is similar to Xpert assay, Xpert Ultra could be used as the initial diagnostic; otherwise, an additional Xpert Ultra assay could be performed after Xpert assay producing a negative outcome to improve case finding.

## MATERIALS AND METHODS

### Ethical approval.

The study was approved by the ethics committees of the three hospitals separately. Because all the samples used were leftover specimens from routine clinical examinations, written informed consent of the patient was waived.

### Study design and participants.

Participants were enrolled consecutively from July 2019 to November 2020 in three tertiary hospitals from three different provinces: Beijing Chest Hospital (Beijing, China), Shandong Provincial Chest Hospital (Jinan, Shandong province, China), and Fuzhou Pulmonary Hospital of Fujian (Fuzhou, Fujian province, China). The TB incidence rate was about 35/100,000, 26/100,000 and 33/100,000 in 2020 in Beijing municipality, Shandong province and Fuzhou province, respectively (according to the Chinese infectious diseases reporting system). Based on the purpose of the test, two different recruitment criteria were applied. The TB case detection group, recruited patients with presumptive pulmonary TB, defined as a case presenting symptoms or signs suggestive of pulmonary TB as per the standard criteria of WHO (22); administered anti-TB drug for ≤3 days in the past 6 months; with more than 5 mL sputum. For the RIF resistance detection group, patients with smear positive sputum and enough volume were recruited consecutively, regardless of their anti-TB treatment status. Patients in the RIF resistance detection group overlapped partially with the case detection study but also included retreated patients or relapse TB cases. Each sputum specimen was processed with smear, culture, Xpert, and Xpert Ultra assay, simultaneously. Drug susceptibility testing (DST) and *rpoB* gene sequencing was conducted for all of the isolates recovered in the RIF resistance detection group.

### Patient categories.

Definite pulmonary TB, defined as microbiologically confirmed TB, with positive smear and/or culture outcome excluding nontuberculous mycobacteria (NTM). Probable TB, defined as neither smear nor culture, was positive, the patient was clinically diagnosed as TB based on clinical findings, radiologic imaging, other molecular tests, or the response to empirical anti-TB treatment. Non-TB indicated that the cases were diagnosed as other diseases, or that the laboratory testing was not suggestive of TB, and the patient improved without receiving antituberculosis treatment. The drug susceptibility status of the patient was referred to the phenotypic DST outcomes.

### Smear and culture.

Direct smear was prepared and stained with auramine and examined by light-emitting diode microscopy. After processing with NALC/NaOH and centrifugation, the resuspended sputum pellet was subjected to culture in a liquid medium using the MGIT 960 system (BD Diagnostic Systems, NJ, USA). For all the isolates, MPT64 antigen testing was performed to confirm the presence of *M. tuberculosis* (Mtb) complex.

### Xpert and Xpert Ultra.

The Xpert and Xpert Ultra assays were performed as per the manufacturer’s instructions. Briefly, 1 mL sputum specimen was mixed with 2 mL sample reagent, vortexed for at least 10 s, and incubated at room temperature for 15 min. A total of 2 mL from the mixture was transferred into the cartridge and loaded into the GeneXpert instrument (Cepheid Inc., Sunnyvale, CA, USA). The automatic detection procedure was then run. For an invalid result, a repeat Xpert and/or Xpert Ultra test was performed on the same sample. Semiquantitative estimation of the Mtb load was also determined by Xpert Ultra as high, medium, low, very low, or trace, depending on the cycle threshold.

### Drug susceptibility testing.

Culture positive samples were subjected for phenotypic DST using the Bactec MGIT 960 system (BD Diagnostic Systems, NJ, USA). The critical concentration of 1 mg/mL was used for RIF.

### *rpoB* gene sequencing.

Isolates were further analyzed by sequencing of an internal region of the *rpoB* gene, which included the RIF resistance-determining region (RRDR), in order to identify mutations associated with RIF resistance. The *rpoB* gene was amplified using a previously published method (23). DNA sequences were analyzed and compared with the sequence of the Mtb reference strain H37Rv using Lasergene software version 7.1.

### Statistical analyses.

The sensitivity, specificity, positive predictive value, and negative predictive value of different assays were calculated against the reference standard. The McNemar’s test was used to compare the sensitivity and specificity of Mtb or RIF detection between Xpert and Xpert Ultra. Statistical analysis was performed using SPSS version 19.0. Differences were considered statistically significant at *P* < 0.05.
